# Nanocomposite-based dual enzyme system for broad-spectrum scavenging of reactive oxygen species

**DOI:** 10.1038/s41598-021-83819-4

**Published:** 2021-02-22

**Authors:** Marko Pavlovic, Szabolcs Muráth, Xénia Katona, Nizar B. Alsharif, Paul Rouster, József Maléth, Istvan Szilagyi

**Affiliations:** 1 Department of Colloid Chemistry, Max Planck Institute of Colloids and Interfaces, 14476 Potsdam, Germany; 2 MTA-SZTE Lendület Biocolloids Research Group, Interdisciplinary Excellence Centre, Department of Physical Chemistry and Materials Science, University of Szeged, 6720 Szeged, Hungary; 3 MTA-SZTE Lendület Epithelial Cell Signaling and Secretion Research Group, Interdisciplinary Excellence Centre, University of Szeged, 6720 Szeged, Hungary; 4 Institute of Condensed Matter and Nanosciences-Bio and Soft Matter, Université Catholique de Louvain, 1348 Louvain-la-Neuve, Belgium

**Keywords:** Materials chemistry, Medicinal chemistry, Physical chemistry

## Abstract

A broad-spectrum reactive oxygen species (ROS)-scavenging hybrid material (CASCADE) was developed by sequential adsorption of heparin (HEP) and poly(L-lysine) (PLL) polyelectrolytes together with superoxide dismutase (SOD) and horseradish peroxidase (HRP) antioxidant enzymes on layered double hydroxide (LDH) nanoclay support. The synthetic conditions were optimized so that CASCADE possessed remarkable structural (no enzyme leakage) and colloidal (excellent resistance against salt-induced aggregation) stability. The obtained composite was active in decomposition of both superoxide radical anions and hydrogen peroxide in biochemical assays revealing that the strong electrostatic interaction with the functionalized support led to high enzyme loadings, nevertheless, it did not interfere with the native enzyme conformation. In vitro tests demonstrated that ROS generated in human cervical adenocarcinoma cells were successfully consumed by the hybrid material. The cellular uptake was not accompanied with any toxicity effects, which makes the developed CASCADE a promising candidate for treatment of oxidative stress-related diseases.

## Introduction

Hybrid materials containing enzymes confined in nano-sized objects or other self-assembled structures have attracted widespread contemporary interest due to their great potential as efficient biocatalytic systems in biomedical and industrial processes^1–3^. Natural enzymes inherently suffer from structural and functional sensitivity to environmental effects leading to a narrow window of operational conditions such as pH and temperature. To counter such drawbacks, enzyme immobilization has been a major focus of several research bodies in the past decades^4–6^. A more recent emphasis has been on co-immobilization to prepare confined enzyme cascades owing to the numerous possible cascade combinations. Among them, antioxidant enzyme cascades immobilized in/on various substrates represent an important class of materials that help combatting oxidative stress^7^ caused by the extensive production of reactive oxygen species (ROS)^8–10^.

ROS are by-products of oxygen metabolism inducing irreversible functional alterations or even complete destruction of vital cell constituents such as lipids, nucleic acids and proteins leading to the development of various diseases^11–13^. One of the most common ROS that are produced in the mitochondrial respiratory chain is superoxide radical anion. Superoxide dismutase (SOD) is a naturally occurring enzyme and it is the primary defense line against these radicals^14^. However, decomposition of superoxide radicals by SOD generates hydrogen peroxide (together with molecular oxygen), another harmful ROS. In a living cellular environment, hydrogen peroxide is consumed by catalase (CAT)^15^ and peroxidases^16^ (e.g., horseradish peroxidase (HRP), chloroperoxidase (CPO) or lactoperoxidase (LPO)) enzymes. Therefore, the eventual combination of SOD with these enzymes gives a promising hybrid system and the cascade reaction can effectively reduce oxidative stress.

In this way, antioxidant enzymes were co-immobilized and cascade systems based on various solid supports were developed. Simultaneous encapsulation of polyelectrolyte-grafted SOD and CAT into hollow silica nanospheres prepared by a microemulsion-based sol–gel templating process was achieved^17^. The obtained hybrid material showed remarkable activity in the decomposition of superoxide radicals and hydrogen peroxide both in test tube reaction and cellular environment. In another contribution, polymeric vesicles were loaded with SOD and CAT leading to cascade decomposition of superoxide radicals^8^. However, diffusion of hydrogen peroxide into these vesicles was limited. In addition, an artificial peroxisome composed of SOD and CAT in a polymeric nanocompartment was developed to combat oxidative stress in cells with significant success^18^.

Concerning the co-immobilization of SOD and peroxidase enzymes, a novel anticancer hydrogel containing SOD and CPO was prepared and converted ROS in tumor cells to singlet oxygen to achieve antiproliferation^10^. In this manner, it is possible to increase the enzyme and substrate concentration inspired by the nature of living cells. A catalytic system was formed by encapsulating SOD and LPO in the cavities of polymeric nanovesicles, which effectively transformed superoxide radicals to water and molecular oxygen^9^. Furthermore, SOD and HRP enzymes were covalently grafted to synthetic dendronized polymer chains and it was confirmed that both enzymes remained active upon immobilization^19^. In a follow-up research, the effect of size of the dendronized polymers on the catalytic activity of the enzyme cascade was assessed^20^.

**Scheme 1:**
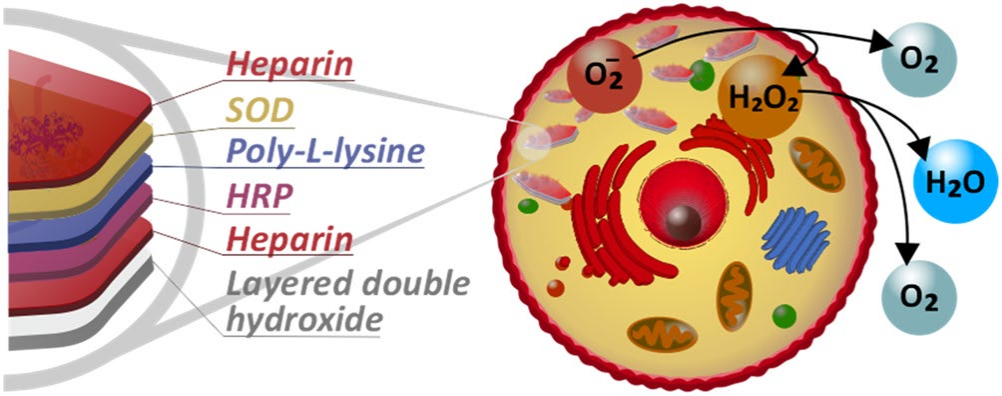
Illustration of the structure of the developed CASCADE nanocomposite and its ROS scavenging ability.

These examples show that the co-attachment of antioxidant enzymes to the same carrier is possible and remarkable activities can be achieved in decomposition of both superoxide radical anions and hydrogen peroxide with the same material. However, the synthetic methods often involved complicated reactions and hardly accessible building blocks, which may impede the potential mass production of the biocatalytic systems. A facile and economic method is proposed here to co-immobilize SOD and HRP enzymes between polyelectrolyte layers built-up on layered double hydroxide (LDH) nanoparticles (Scheme 1). LDHs are versatile clay materials^21–24^ of several advantages such as cytocompatibility, sufficient anion-exchange capacity and ease of synthesis even in larger amounts, which make LDHs to be very efficient delivery agents in biomedical applications^25–27^. The structural, colloidal and functional stability were investigated by various techniques and the ability to reduce intracellular oxidative stress was assessed. The expected role of the nanoparticulate support was to achieve high stability, to confine the enzymes similar to the intracellular environment and to enhance enzyme penetration through the cell wall.

## Results

### Design of the antioxidant composite

**Figure 1:**
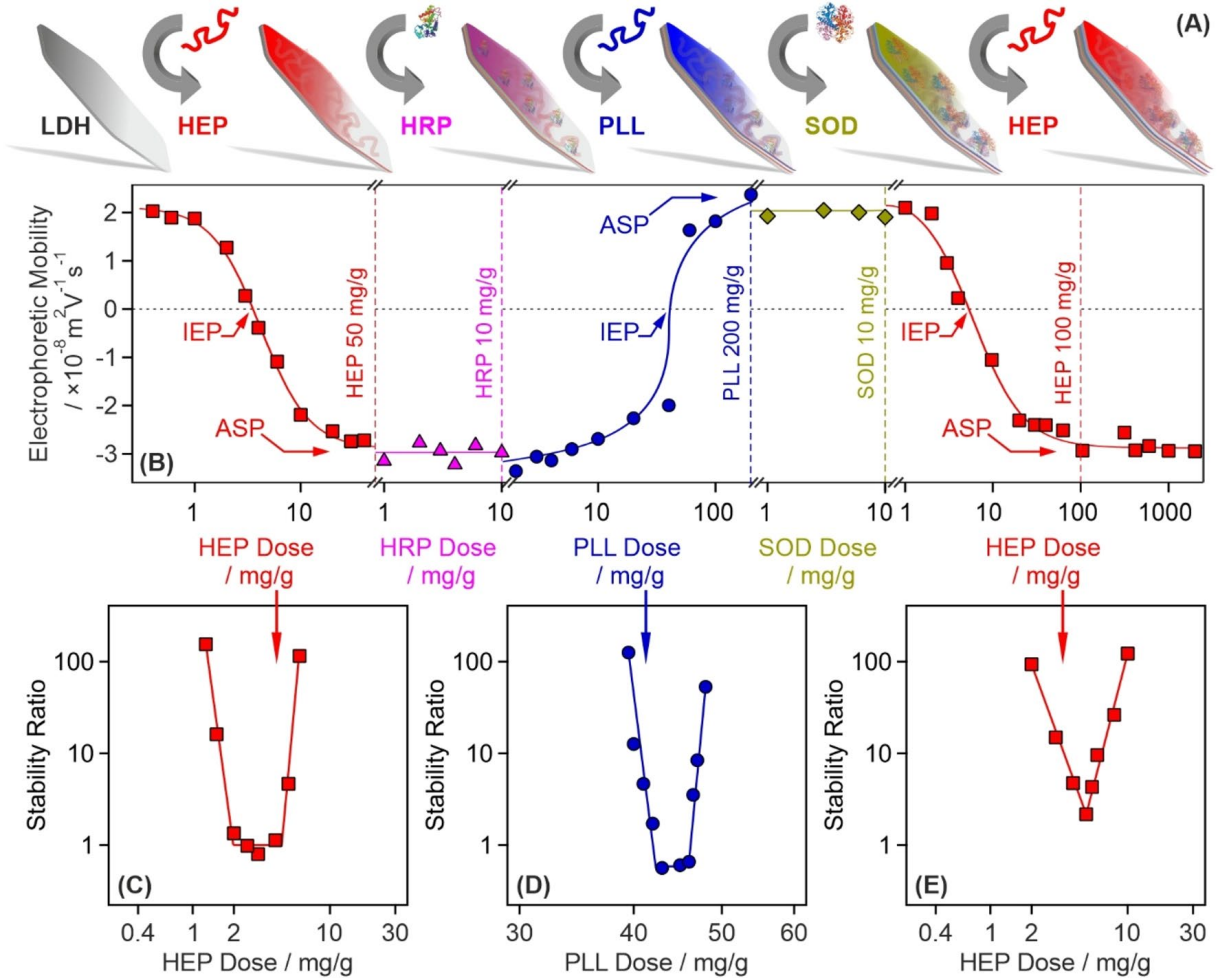
Illustration of the sequential adsorption process and tendencies in the charging and aggregation properties during the build-up of the polyelectrolyte-enzyme layers on the LDH particles. **(A)** Schematic representation of the preparation of the final CASCADE composite. **(B)** Changes in electrophoretic mobilities during the sequential adsorption procedure illustrated in **(A)**. The dashed vertical lines show the doses used in the synthesis of CASCADE. **(C)** Stability ratio of LDH particles as a function of the HEP dose. **(D)** Stability ratio of LDH–HEP–HRP particles as a function of the PLL dose. **(E)** Stability ratio of LDH–HEP–HRP–PLL–SOD particles as a function of the HEP dose. Note that the mg/g unit refers to mg of polyelectrolyte or enzyme per one gram of LDH particles. The solid lines are just to guide the eyes. IEP and ASP are abbreviations of doses corresponding to the isoelectric point and to the onset of the adsorption saturation plateau, respectively. The electrophoretic mobilities were determined with ± 10^–9^ m^2^ V^−1^ s^−1^ precision, while the average error of the stability ratios is 10%.

The polyelectrolyte-based sequential adsorption method^28^ on LDH nanoclay support was applied to co-immobilize SOD and HRP enzymes for tandem decomposition of superoxide radical anions and hydrogen peroxide (Fig. 1A). First, the LDH carrier was prepared by the co-precipitation technique and subsequent hydrothermal treatment^29^ to improve the size distribution and polydispersity of the particles. The synthetic protocol is given in the Supplementary Information (SI). The formation of LDH was proven by X-ray diffraction (XRD) and Infrared (IR) spectroscopy. The assignment of the Miller indices, shown in Fig. S1A (in the SI), unambiguously confirms the existence of the lamellar structure, while the IR spectrum (Fig. S1B) of the obtained material contained the characteristic vibrations of the intralamellar metal hydroxide groups (552, 777 and 941 cm^−1^) and the interlamellar carbonate anions (665 and 1351 cm^−1^). More details about the structural characterization of the LDH are given elsewhere^30^.

Based on previous experiences with LDH-polyelectrolyte systems^30–32^, the surface charge and particle aggregation were tuned in each step of the sequential adsorption process to obtain stable particle dispersions. This issue was rarely addressed in earlier reports dealing with the co-immobilization of antioxidant enzymes. However, lack of sufficient colloidal stability, i.e., formation of particle aggregates, during preparation leads to several disadvantageous features in the final material such as decreased surface area, limited diffusion of the substrates to the active centers and phase separation due to formation of higher sized clusters.

The tendencies in surface charges in the individual steps were followed in electrophoretic mobility measurements (Fig. 1B). Accordingly, heparin (HEP) adsorption on the oppositely charged LDH significantly altered the surface charge, which was neutralized at the isoelectric point (IEP) and reversed at appropriately high HEP doses. Such a charge reversal was also reported in other LDH-polyelectrolyte systems^30,32,33^ and originate mainly from entropy gain^34^, hydrophobic interactions^35^ and ion correlations forces^36^. The surface saturation was indicated by a plateau (ASP) in the mobilities at high doses. As it was pointed out earlier^30^, intercalation of HEP between the LDH layers is not possible due to the limited interlayer space. Adsorption of HRP of positive overall charge did not change the charge of LDH–HEP (HEP dose was set at the ASP) significantly due its limited amount and low charge density. To optimize the conditions for SOD immobilization, the negatively charged LDH–HEP–HRP surface was modified with poly(l-lysine) (PLL), which possesses positive charge and thus, charge neutralization and reversal occurred similar to the first step involving LDH and HEP. Characteristic IEP and ASP values were observed with the PLL too. The LDH–HEP–HRP–PLL was then decorated with SOD, nevertheless, the electrophoretic mobilities were the same within the experimental error at the enzyme dose range investigated. Finally, the LDH–HEP–HRP–PLL–SOD composite was coated with a terminating HEP layer making the final LDH-HEP–HRP–PLL–SOD–HEP (henceforth denoted as CASCADE) material negatively charged. The negative sign of charge is usually beneficial to hinder protein corona formation in biofluids^37^ and does not prevent the nanoparticle penetration through the like-charged cell wall^38^. In CASCADE, the polyelectrolyte doses were set at the ASP (HEP1: 50 mg/g, PLL: 200 mg/g and HEP2: 100 mg/g) and the doses of both SOD and HRP were adjusted to 10 mg/g. These conditions resulted in a hydrodynamic radius of (121.8 ± 3.5) nm, polydispersity index of (0.35 ± 0.08) and electrophoretic mobility of (− 2.9 ± 0.1) × 10^–8^ m^2^ V^−1^ s^−1^ determined for CASCADE in stable dispersions at 10 mg/g particle concentration.

As mentioned above, remarkable colloidal stability of the sub-systems during the sequential adsorption process is crucial to obtain stable dispersions of primary CASCADE particles. Therefore, stability ratios were measured by dynamic light scattering (DLS) in the individual systems to probe possible particle aggregation. Note that stability ratios of unity refer to rapid aggregation and unstable dispersions, while higher values indicate slower aggregation and more stable samples^39,40^. The theoretical and technical backgrounds of the stability ratio measurements are given in the SI.

Using the same experimental conditions as in the mobility study, the effect of polyelectrolyte or enzyme adsorption on the stability ratios was determined. In the LDH–HEP system, stability ratios close to one indicated rapid particle aggregation and unstable dispersions near the IEP, while highly stable colloids were obtained at low and high HEP doses (Fig. 1C). No measurable particle aggregation was detected at the dose applied in CASCADE. Similar U-shaped stability ratio curves were measured with PLL (Fig. 1D) and the second HEP (Fig. 1E) layer, i.e., the particles rapidly aggregated close to the IEP, while saturated polyelectrolyte layers formed at the ASP stabilized the dispersions. Such charge-aggregation patterns have been reported earlier with LDH-polyelectrolyte systems^32,33^ and qualitatively agree well with the classical theory developed by Derjaguin, Landau, Verwey and Overbeek (DLVO)^41^.

Concerning the influence of enzyme adsorption on the colloidal stability of the samples, the electrophoretic mobilities of LDH-HEP did not change significantly in the 1–100 mg/g HRP concentration range (Fig. S2A). They increased at higher doses, however, this regime is out of the scope of the present investigation. The same conclusions can be made based on the mobility data recorded for LDH–HEP–HRP–PLL in the presence of SOD (Fig. S2B). In addition, the hydrodynamic radii of the particles did not change with time at 10 mg/g enzyme doses (Fig. S2 insets) indicating a highly stable dispersion under these experimental conditions.

On the basis of the results of the above electrophoretic and light scattering measurements one can conclude that both CASCADE and its sub-systems form highly stable dispersions, once the optimized doses (HEP1: 50 mg/g, HRP: 10 mg/g, PLL: 200 mg/g, SOD: 10 mg/g and HEP2: 100 mg/g) are applied during the sequential adsorption process. This information is particularly important for the successful application of the composite to combat ROS.

### Structural and colloidal characterization of CASCADE

**Figure 2:**
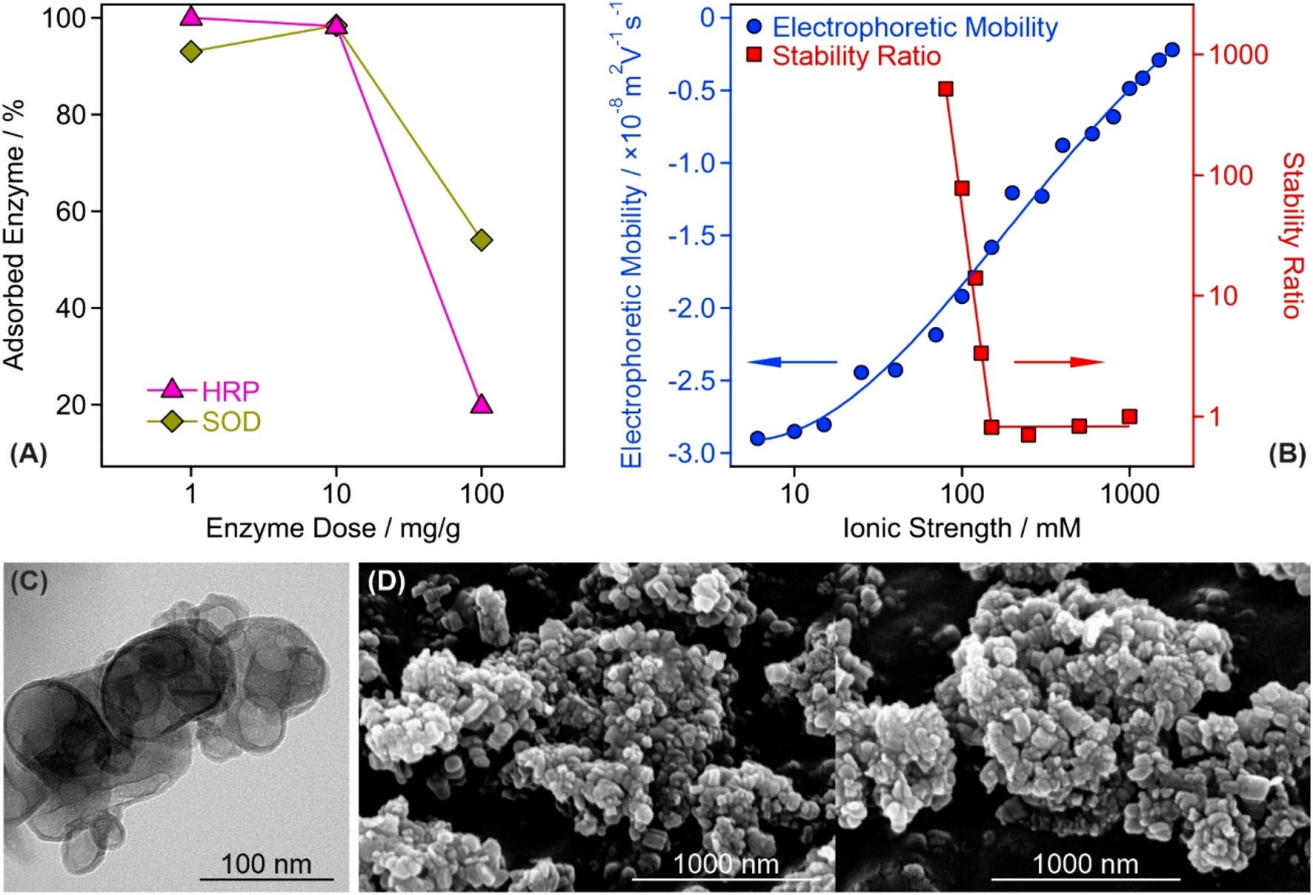
Structural and colloidal features of the CASCADE composite. **(A)** Partitioning of HRP and SOD enzymes between the bulk and the surface at different enzyme doses. The data indicate that the majority of the added enzyme molecules is adsorbed on the particle surfaces at 10 mg/g dose. **(B)** Influence of the ionic strength (adjusted by NaCl) on the electrophoretic mobility and stability ratio values of the CASCADE particles. For the latter, the critical coagulation concentration, the threshold value, which separates fast and slow aggregation regimes, was found to be 150 mM. **(C)** TEM and **(D)** SEM images of the obtained hybrid material. In **(A,B)** the solid lines serve to guide the eyes.

The enzyme doses were selected on the basis of charging and aggregation behavior of the composite particles, however, the nature of enzyme immobilization, i.e., whether their adsorption is quantitative or partitioning takes place between the surface and the bulk solution, must be studied in order to assess the structural stability of CASCADE. This issue was addressed by performing the Bradford test as a typical spectrophotometric method that can detect enzymes in a solution (see SI for further details of this Bradford test)^42,43^. Three different doses (1, 10 and 100 mg/g) were probed for both SOD and HRP enzymes. It was found that 93.0 ± 2.8%, 98.4 ± 3.0% and 54.1 ± 1.6% of the total added amount of SOD enzyme was immobilized in CASCADE at 1, 10 and 100 mg/g enzyme doses, respectively. For HRP, these number were 99.9 ± 3.0%, 98.3 ± 2.9% and 19.8 ± 0.6% (Fig. 2A). These results clearly indicate that complete adsorption occurred at the lower doses, i.e., no enzyme was found in the surrounding solution at 1 and 10 mg/g loadings. For the 100 mg/g loading, in contrast, about 46 and 80% of the total SOD and HRP, respectively, remained in the bulk confirming the partitioning of the enzymes between the surface and the solution. Therefore, these results unambiguously support that the total amounts of added enzymes are embedded in the CASCADE hybrid at 10 mg/g enzyme doses.

Ionic strength is an important parameter in potential applications of antioxidant nanoparticles, since the presence of high level of electrolytes is one of the most frequent reasons for particle aggregation for materials including LDHs^32,44^. This may lead to significant loss in the ROS-scavenging activity. Therefore, the resistance against salt-induced aggregation was studied in electrophoretic and DLS measurements. In general, the colloidal stability of the CASCADE particles in salt solutions of different concentrations can be described by the DLVO theory^40,41,44^. Accordingly, stability ratios decreased by increasing the ionic strength (Fig. 2B) due to the weakening of the repulsive double layer forces, which vanish at high salt levels and thus, attractive van der Waals interactions predominate giving rise to rapid particle aggregation. This hypothesis is supported by the tendency in the mobility values, whose magnitude decreased with increasing the ionic strength due to the shrinking of the electrical double layer by progressive salt screening.

The sudden transition between the fast and slow aggregation regimes occurred at the so-called critical coagulation concentration, which was determined to be 150 mM for CASCADE. This value is 6-times higher than the one reported for the bare LDH nanoclay^31^ indicating an enormous stabilizing effect of the polyelectrolyte-enzyme layer formed on the LDH surface.

The morphology of the CASCADE material was also investigated by recording transmission (TEM, Fig. 2C) and scanning (SEM, Fig. 2D) electron microscopy images. For comparison, the TEM and SEM images of the bare LDH support are shown in Fig. S3A and S3B-C, respectively. It can be concluded that the polyelectrolyte and enzyme coating had no impact on the sample morphology. The particles possessed disc-like shape, which is typical for LDH nanoclays prepared by the co-precipitation method and treated hydrothermally thereafter^29^. Note that CASCADE aggregates are present in the TEM/SEM images due to the sample preparation process during imaging. This includes dispersion drying leading to the formation of clusters of particles upon evaporation of the solvent.

### Enzymatic activity of CASCADE

**Figure 3:**
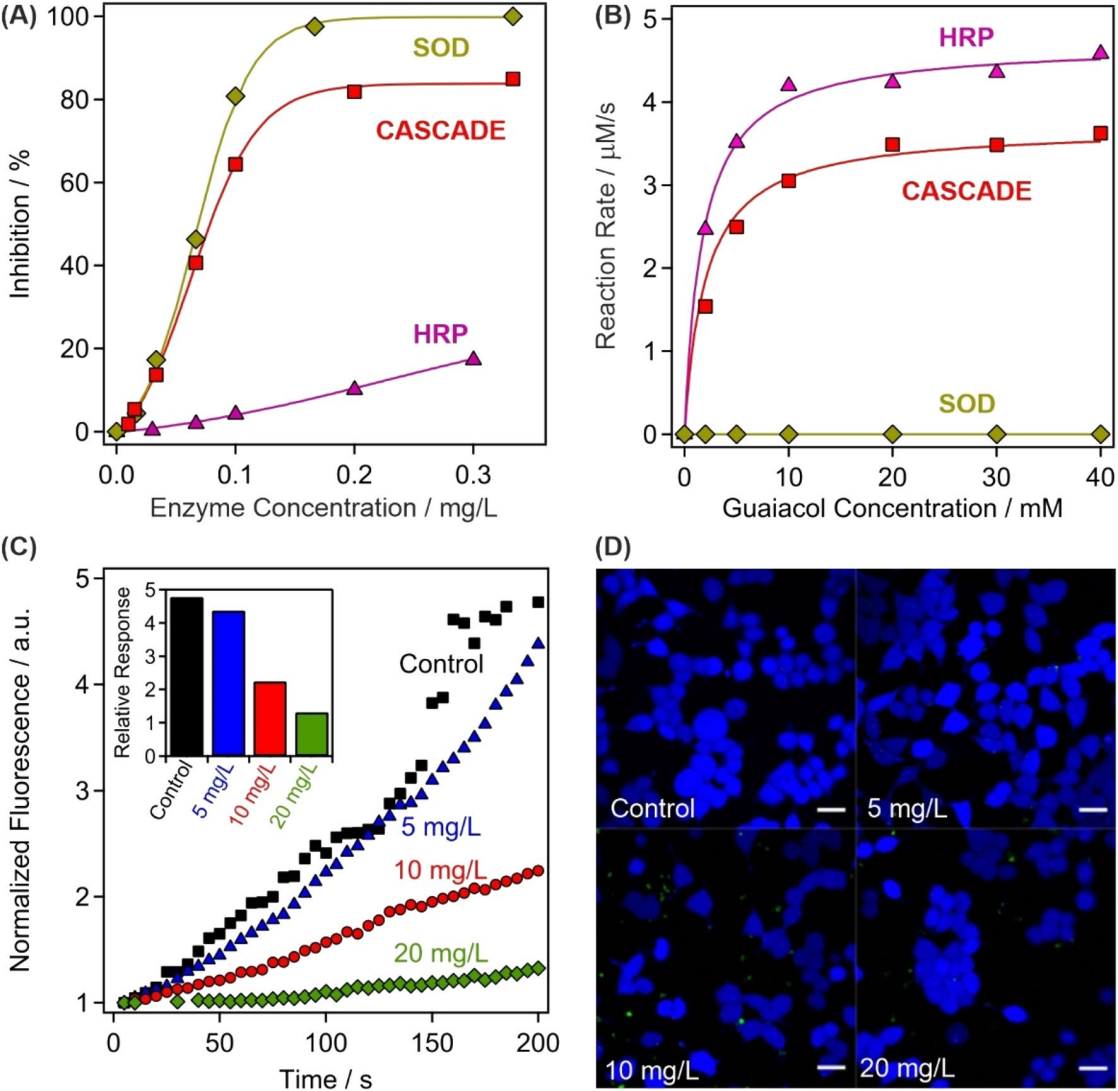
Assessment of the antioxidant activity of CASCADE. **(A)** SOD-like activity expressed by the inhibition of the NBT-superoxide radical reaction. The lines are mathematical functions used to interpolate the IC_50_ values. **(B)** HRP-like activity measured in the guaiacol-hydrogen peroxide reaction, where CASCADE acts as a catalyst. The solid lines represent the fits using the Michaelis–Menten model. **(C)** The fluorescent intensity of H_2_DCFDA dye over time at different CASCADE loadings into HeLa cells. The maximal relative response of the detected fluorescent intensity is shown in the inset and in Table S1. **(D)** Live (blue fluorescent) HeLa cells were observed during the control experiments and at 5, 10 and 20 mg/L CASCADE doses. Scale bars represent 20 μm dimension.

The obtained composite was first tested in biochemical test reactions to assess the SOD and HRP activities. The SOD assay^45^ is based on the enzymatic production of superoxide radicals, whose presence can be detected upon reaction with NBT (nitroblue tetrazolium). This reaction is inhibited by the SOD enzyme and its concentration necessary to achieve 50% inhibition is called IC_50_ value, which can be read from the inhibition versus enzyme concentration plots (Fig. 3A). Detailed descriptions of the SOD assay are given in the SI. The IC_50_ data are listed for CASCADE together with the values measured for native SOD and HRP in Table 1. Two main conclusions can be drawn from these experimental data. First, the activity of CASCADE and SOD are very similar, which is a strong proof for the preservation of the enzyme structural integrity upon immobilization. Second, HRP does not show significant SOD activity, i.e., the IC_50_ value could not be determined, in the concentration regime investigated.

**Table 1:** Enzymatic activity and kinetic parameters of CASCADE and the native enzymes.

Sample	SOD activity	HRP activity		
	IC_50_ (mg/L)	v_max_ (mM/s)	K_M_ (mM)	k_cat_/K_M_ (1/Ms)
SOD (native)	0.069 ± 0.003	N/A^a^	N/A^a^	N/A^a^
HRP (native)	N/A^a^	0.0046 ± 0.0002	1.56 ± 0.08	(2.59 ± 0.13) × 10^5^
CASCADE	0.078 ± 0.004	0.0038 ± 0.0002	2.64 ± 0.13	(1.27 ± 0.06) × 10^5^

The average error of the assay measurements is about 5%.

^a^The enzyme was inactive, i.e., the enzymatic activity could not be determined.

The HRP assay^46^ is based on the catalytic oxidation of guaiacol, being the substrate, in the presence of hydrogen peroxide (see SI for more details). Comparing the kinetic data shown in Fig. 3B, it is evident that native SOD did not show any activity in the assay. In the case of CASCADE and HRP, the maximum reaction rate of the guaiacol oxidation (v_max_) was lower for CASCADE, although the substrate concentration at half of the maximum reaction rate (K_M_) remained comparable. The turnover number (k_cat_) was also calculated enabling the determination of the catalytic efficiency (k_cat_/K_M_). This is an important parameter that gives information about the initial rate of reaction at substrate concentrations that are significantly lower than K_M_, similar to the physiological conditions. A twofold drop in the catalytic efficiency occurred upon HRP immobilization and coating, nevertheless, the efficiency remained reasonably high making CASCADE suitable for hydrogen peroxide consumption.

The above results clearly show that both SOD and HRP enzymes remained highly active upon immobilization. Since enzyme immobilization may result in complete or significant loss in the activity, the unchanged performances are great achievements of the present work.

### Cellular enzymatic activity and cytocompatibility

The above discussed enzymatic activity of CASCADE was also assessed in cellular environment. The uptake by HeLa cells was proved by a guaiacol assay-inspired method after lysis, which is detailed in the SI. After incubating the cells with CASCADE at 5 mg/L concentration, the colored product from guaiacol was not detectable, possibly due to the low total concentration of CASCADE taken-up by the cells (Fig. S4). However, at 10 and 20 mg/L loadings, the formation of the oxidized guaiacol was clearly detected by the increase in the absorbances, which was proportional to the CASCADE content. It was shown earlier that no enzyme leakage occurs at the dose applied in the timeframe of the experiment (Fig. 2A), therefore, one can claim that the CASCADE system successfully enters the cells after 60 min incubation.

Besides, fluorescent measurements were carried out using 2′,7′-dichlorodihydrofluorescein diacetate (H_2_DCFDA) dye to quantify the intracellular ROS-scavenging activity of CASCADE. The H_2_DCFDA is indirectly oxidized by ROS and the developed product generates the fluorescence signal (more details are given in the SI). The ROS production was artificially increased by adding 50 μM menadione to the samples that generates ROS through redox cycling and triggers cell death at high concentrations. This method has been proven as sufficient toll to assess oxidative stress reducing ability of antioxidant compounds^47,48^. For comparison, a control experiment was performed without adding any biocatalytic compounds. After the 2 min initial perfusion with HEPES buffer only, the increase of intracellular oxidative stress was apparent via perfusion with HEPES mixed with menadione (Fig. 3C). The experiment was repeated with the cells incubated with CASCADE at 5, 10 and 20 mg/L concentrations for 60 min. While at 5 mg/L a slight decrease in the intracellular oxidative stress was already observable, the effect became more pronounced at 10 and 20 mg/L, where significant decrease in the fluorescent intensity was apparent. Note that CASCADE and the dye molecules were incubated for 20 min prior to the experiments (i.e., before menadione was added) and no fluorescence signal was detected.

The data were analyzed with the statistical Mann–Whitney U-test (Fig. 3C inset). Applying a significance level (p value) of 5% for the test, the intensity of ROS production in 5 mg/L CASCADE incubated cells is not significantly different from the control group. However, at 10 and 20 mg/L loadings, the signals are remarkably lower compared to the control measurement with a p value under 0.01%. The full set of data is shown in Table S1. One can conclude that at 5 mg/L dose, even with proper enzymatic activity, the amount of immobilized enzymes taken-up was not sufficient to prevent intracellular oxidative stress. In contrast, at higher incubation doses, the antioxidant effect was expressed. Note that fluorescent intensity is already observable in the initial period without added menadione, since ROS are self-generated by the cells when removed from their feeding media. However, this initial fluorescence is also reduced at 10 and 20 mg/L CASCADE content, indicating the antioxidant effectiveness of the material.

On the one hand, the reduced amount of ROS in the cells originated from the preserved catalytic activity of the immobilized enzymes. However, ROS are not generated if cellular respiration ceases, i.e., if the cell death occurs by apoptosis or necrosis. Therefore, apoptosis/necrosis kit tests (see SI for details) were performed to measure the cell viability at different CASCADE doses (Fig. 3D). Note that the healthy cells are blue, while apoptosis and necrosis cause color change to green and red, respectively. However, cell death was not detected up to 20 mg/L CASCADE loading proving the remarkable activity of the immobilized HRP and SOD enzymes. Extensive apoptosis was observed at higher dose, as shown for 40 mg/g loading in Fig. S5. These results demonstrate the cellular uptake and cytocompatibility of the developed antioxidant material, while also maintaining its outstanding enzymatic activity in scavenging of biologically induced ROS in HeLa cells.

In conclusion, LDH nanoclays were synthesized and used as solid support for enzyme immobilization. The sequential adsorption method was suitable to build polyelectrolyte-enzyme layers on the LDH particles, however, the colloidal stability of the sub-systems prepared in the synthetic steps had to be optimized by tuning the surface charge and aggregation processes. HEP and PLL polyelectrolytes have proven to be appropriate building blocks, by strongly interacting with the oppositely charged particle surfaces leading to the formation of saturated polyelectrolyte layers and to an improved colloidal stability of the hybrid material. HRP and SOD enzymes were successfully embedded between the polyelectrolyte layers, since their strong interaction with the HEP and PLL chains prevented their desorption from the surface and provided an excellent structural stability. Remarkable enzymatic activity was observed in standard assays in dismutation of superoxide radical anions and in consumption of hydrogen peroxide. The preserved enzymatic activity was also confirmed by in vitro experiments. The CASCADE material was taken up by HeLa cells within reasonable incubation time. They showed sufficient cytocompatibility and remarkably decomposed the artificially generated ROS, when the CASCADE loading was appropriately high. Summarily, a facile synthetic route was demonstrated to obtain an effective antioxidant bionanomaterial composed of naturally occurring and easily accessible building-blocks. The obtained CASCADE hybrid possessed excellent structural, colloidal and functional stability, which makes it a promising candidate in antioxidant treatments of inflammatory bowel diseases via rectal injection.

## Methods

### Materials

The quality and the source of all the chemicals are listed in the SI and they were used as received, unless stated otherwise.

### Electrophoresis

The electrophoretic mobilities were determined on a ZetaSizer Nano ZS instrument (Malvern) that utilizes a He–Ne laser of 632 nm wavelength and applies the phase analysis light scattering technique. All samples were prepared the day prior to the measurement in order to equilibrate overnight. The final LDH concentration in the dispersions was set to 10 mg/L, while the final volume was 5 mL. Measurements were carried out in plastic capillary cells (Malvern) and the average value of five separate measurements was reported. This protocol led to a mean error of 5%.

### Light scattering

The hydrodynamic radii were measured by DLS using a CGS-3 goniometer system (ALV) at 90° scattering angle. The correlation function was collected for 20 s and the cumulant fit was used to obtain the diffusion coefficient, which was applied in the Stokes–Einstein equation to calculate the hydrodynamic radius^49^. All aggregation tests were performed in round borosilicate test tubes (Kimble Chase), with a final sample volume of 2 mL and particle concentration of 10 mg/L.

### Structural analysis

Morphological studies were carried out on a Hitachi S-4700 SEM and TEM at various magnifications using 10 kV (SEM) and 200 kV accelerating voltage (TEM). Before SEM measurements, the samples were sputter coated with gold. XRD and IR spectroscopy measurements were performed with an Empyrean diffractometer (Panalytical) and a Spectrum 100 FT-IR spectrometer (PerkinElmer), respectively.

### Determination of the enzyme content and enzymatic assays

The concentration of the enzymes in solution was measured with the standard Bradford test^42^. The SOD and HRP activities were determined by the Fridovich^45^ and guaiacol^46^ assays with an average error of 10% and 5%, respectively. Detailed descriptions of these protocols are given in the SI.

### Cellular uptake

The CASCADE material was added to HeLa cells in a culture flask of 70% confluency covered with 1 mL feeding media in various concentrations and the mixtures were incubated for 60 min (37 °C, 5 v% CO_2_ in air). After that, the liquid was removed, the cells were washed with Dulbecco’s PBS (phosphate buffered saline, 9.5 mM phosphate content) twice to remove any residual CASCADE and the cells were lysed with RIPA (radioimmunoprecipitation assay, tenfold dilution) lysis buffer for 30 min. Each 10 mL of lysis buffer contained one mini cOmplete ULTRA tablet, a protease inhibitor. Cellular debris were removed via centrifugation at 4 °C (13,000 rpm, 10 min) and the supernatant liquid obtained was investigated by a guaiacol assay as described in the SI.

### Cellular ROS detection

After the incubation step described above, a ROS sensitive dye (H_2_DCFDA, 5 μM concentration) in HEPES buffer solution was added to the cells. Microscopic analyses were carried out by following the increase in the drift corrected green fluorescence response of the ROS-sensitive dye on an LSM 880 confocal microscope (Zeiss) with a standard ROS detecting procedure detailed in the SI. Menadione in HEPES buffer (see SI for exact composition) was used to generate intracellular ROS. For further data evaluation, the Mann–Whitney U-test was performed in GraphPad Prism software package version 8.0.0 for Windows (GraphPad).

### Cell viability study

Apoptosis/necrosis assay kit (blue, green and red) was used to monitor the live (blue), apoptotic (green) and necrotic cells (red) after 60 min incubation (HeLa cells fixed on cover glass with 70% confluency under 1 mL feeding media, 37 °C, 5 v% CO_2_ in air) with the composite material at various concentrations. The measurements were carried out on the same microscope as the ROS experiments according to a standard procedure detailed in the SI. An average error of 10% must be considered for all data obtained from the above activity and viability measurements.

## Supplementary Information

Supplementary Information.

## Data Availability

The data that support the findings of this study are available from the corresponding author upon reasonable request.
