# Supplementary material for: Nanocomposite-based dual enzyme system for broad-spectrum scavenging of reactive oxygen species
Source: Sci Rep. 2021 Feb 22;11:4321. doi: 10.1038/s41598-021-83819-4 (PMC7900168; doi:10.1038/s41598-021-83819-4)
Supplement: Supplementary file 1 — Supplementary Information. [file 41598_2021_83819_MOESM1_ESM.pdf]

Supplementary Information

Nanocomposite-based dual enzyme system for broad-spectrum scavenging of reactive oxygen species

Marko Pavlovic[1], Szabolcs Muráth[2], Xénia Katona[3], Nizar B. Alsharif[2], Paul Rouster[4], József Maléth[3] & Istvan Szilagyi[2]*

[1]Department of Colloid Chemistry, Max Planck Institute of Colloids and Interfaces, D-14476 Potsdam, Germany

[2]MTA-SZTE Lendület Biocolloids Research Group, Interdisciplinary Excellence Centre, Department of Physical Chemistry and Materials Science, University of Szeged, H-6720 Szeged, Hungary

[3]MTA-SZTE Lendület Epithelial Cell Signaling and Secretion Research Group, Interdisciplinary Excellence Centre, University of Szeged, H-6720 Szeged, Hungary

[4]Institute of Condensed Matter and Nanosciences - Bio and Soft Matter, Université Catholique de Louvain, B-1348 Louvain-la-Neuve, Belgium

*Corresponding author. Email: szistvan@chem.u-szeged.hu

Detailed experimental part

Materials. Horseradish peroxidase (HRP, type VI (EC 1.11.1.7)) and bovine erythrocyte superoxide dismutase (SOD, (EC 1.15.1.1)) were bought from Sigma-Aldrich in the form of salt-free lyophilized powder and used without any further purification. NaCl (99.5%), NaOH (97%), 20 wt% HCl (99%), H_3PO_4 (99.99%), Na_2CO_3 (99.5%), $Mg(NO_3)_2 \times 6H_2O$ (99.99%), $Al(NO_3)_3 \times 9H_2O$ (98%), Na_2HPO_4 (99%), NaH_2PO_4 (99%), 30 wt% hydrogen peroxide (analytical grade), poly-L-lysine hydrochloride (PLL, 15-30 kg/mol), 95% ethanol (analytical grade), KCl (99%), $CaCl_2 \times 2H_2O$ (analytical grade), $MgCl_2 \times 6H_2O$ (99%), glucose (analytical grade), HEPES (analytical grade), menadione (analytical grade), bovine milk xanthine oxidase (XO, (EC 1.17.3.2)), xanthine (99%), nitroblue tetrazolium (NBT, 98%) chloride and guaiacol (98%) were also purchased from Sigma-Aldrich and used as received. Coomassie Brilliant Blue (98%) dye was bought from Thermo Fisher Scientific. Low molecular weight sodium heparin (HEP, 12-15 kg/mol) was obtained from Acros Organics.

Human cervical adenocarcinoma HeLa cells were purchased from ATCC (ATCC-CCL-2) and were stored in liquid nitrogen. When needed, the cells were grown in Dulbecco's modified Eagle's medium (Sigma-Aldrich) alongside with 10% Fetal Bovine Serum (FBS), 1% kanamycin antibiotic, 1% glutaMAX (Gibco) incubated at 37 °C and 5 v% CO_2 in air atmosphere. The cells were routinely grown in 100 mm plastic tissue culture dishes (Greiner). For the cellular experiments, all the materials were of cell biological purity. Dulbecco's PBS was acquired from Lonza, RIPA lysis buffer from Merck, cOmplete ULTRA tablets from Roche, reactive oxygen species (ROS) sensitive dye 2',7'-dichlorodihydrofluorescein diacetate (H_2DCFDA) from Thermo Fisher Scientific. Blue-green-red apoptosis/necrosis full assay kit (ab176749) was purchased from Abcam.

The HEPES buffer used for cell experiments contained physiological components, i.e., NaCl (140 mM), KCl (5 mM), $CaCl_2$ (5 mM), $MgCl_2$ (1 mM) and glucose (10 mM). The HEPES

content was 10 mM and the pH was adjusted to 7.5 with NaOH, if necessary. All the experiments were performed at room temperature and the samples were prepared using ultrapure water (Millipore).

Preparation of the carrier layered double hydroxide. The layered double hydroxide (LDH) material was synthesized by the flash co-precipitation method[1], as detailed elsewhere[2]. Briefly, $Mg(NO_3)_2 \times 6H_2O$ and $Al(NO_3)_3 \times 9H_2O$ were dissolved in 100 mL ultrapure water with a stoichiometric ratio of 2:1. A second solution containing 20 mL of 1 M NaOH and Na_2CO_3 in a 1:1 stoichiometric ratio to $Mg(NO_3)_2 \times 6H_2O$ was prepared. These two solutions were rapidly mixed together and the mixture was stirred for 24 h. The pH of the obtained slurry was monitored and kept at 9. The solid product was filtered on 0.2 μm pore size nylon filters (Millex) and washed thoroughly with ultrapure water. The solid material was dried overnight in an oven at 60 °C prior to dispersing in water to get 4 wt% dispersions. These samples were transferred to an autoclave with PTFE inlet (Col-Int Tech) for hydrothermal treatment for 24 h at 120 °C. The final product was washed several times with ultrapure water and re-dispersed as 1 wt% dispersion that served as stock.

Preparation of CASCADE. 1000 mg/L stock solutions of HPR, SOD, PLL and HEP were prepared, while for LDH, a stock aqueous dispersion with a concentration of 10000 mg/L was used. In the sequential adsorption method, 900 μL of ultrapure water were added first to 100 μL of bare LDH stock and the sample was vortexed for 10 s and left standing for 10 min prior to the addition of 50 μL HEP. The same procedure was repeated with calculated volumes of HRP, PLL, SOD and again HEP stock solutions to achieve the desired composition in **CASCADE** (HEP1: 50 mg/g, HRP: 10 mg/g, PLL: 200 mg/g, SOD: 10 mg/g and HEP2: 100 mg/g). The sample preparation was finalized by adding ultrapure water to reach the final volume of 1350 μL.

Determination of the stability ratio. Time-resolved dynamic light scattering (DLS) measurements were performed to determine stability ratio (W) values. For this, the evolution of the hydrodynamic radius (R_h) was followed in time (t) under different experimental conditions (e.g., ionic strength and polyelectrolyte dose) and W was calculated as follows[3]:

$$W = \frac{k_{app}^{fast}}{k_{app}} = \frac{\frac{1}{R_h(0)}\frac{dR_h(t)}{dt}\Big|_{t\to 0}^{fast}}{\frac{1}{R_h(0)}\frac{dR_h(t)}{dt}\Big|_{t\to 0}} \tag{1}$$

where $R_h(0)$ is the hydrodynamic radius of the primer particle, k_{app} is the apparent aggregation rate constant measured in the actual experiment. In case of k_{app}^{fast} the measurement was performed in 1 M NaCl solutions, where the salt concentration is high enough to completely screen the charge of the particles and to cause fast aggregation. Under this condition, the aggregation is controlled solely by the diffusion of the particles. Therefore, $W = 1$ refers to fast aggregation and unstable samples, while higher values indicate more stable dispersions and slower aggregation process. The average error of the stability ratio data is 10%.

Bradford test. The Bradford test[4] was applied on the supernatant of the dispersions to monitor the adsorbed amount of enzymes. The Bradford reagent was prepared in the classical way, by dissolving 100 mg of Coomassie Brilliant Blue dye in the mixture of 50 mL of 95% ethanol and 100 mL of 85% phosphoric acid. The solution was then completed with water to 1000 mL in a volumetric flask. Initially, standard solutions of SOD and HRP were prepared and the respective calibration curves were plotted ranging from 1 mg/L to 20 mg/L enzyme concentrations and the absorption spectra were recorded with a spectrophotometer. Two characteristic absorption bands occur, namely at 465 nm and 595 nm, that originate from the free dye and both the dye-enzyme complex and free dye, respectively. The difference between these absorption bands was determined and plotted as a function of the enzyme concentration to obtain the calibration curves. Enzyme concentrations in the actual samples were measured with the above protocol

using the calibration curves. These actual samples were obtained for the Bradford test after removal of the nanocomposites from the samples by filtration. The dispersions were left to equilibrate for at least 3 hours prior to filtration and testing. The above protocol gives an average error of 3%.

SOD assay. The classic Fridovich method was employed to determine the enzymatic activity of the hybrid materials and bare SOD[5]. All the solutions for this assay were prepared in phosphate buffer at pH 7.5 with a final phosphate concentration of 1 mM. Each sample was prepared by mixing 0.1 mL of 3 mM NBT, 0.3 mL of 3 mg/mL XO and 0.1 mL of 3 mM xanthine followed by the immediate addition of 0.1-2.5 mL of solution containing the enzyme. The test was followed spectrophotometrically at 565 nm due to the formation of the blue reduced form of NBT owing to the presence of superoxide radical anions produced in the xanthine-XO reaction. In the presence of SOD or SOD-active compound, superoxide radical anions are eliminated by the antioxidant material and thus, the formation of the blue compound is prevented. In this case, the solution maintains a typical pale-yellow color. The increase in the absorbance was followed and recorded over 5 min (ΔA_s), starting from 1 min after mixing all the components. The same increase was measured for the sample in the absence of any enzymatically active compounds (ΔA_0). Finally, the inhibition (I in %) was calculated as follows:

$$\boldsymbol{I} = \frac{\Delta \boldsymbol{A_s} - \Delta \boldsymbol{A_0}}{\Delta \boldsymbol{A_0}} \tag{2}$$

By plotting the inhibition values as a function of the enzyme concentration, the enzyme content that causes 50% of the maximum inhibition was determined. This is the so-called IC_{50} value, which is used to compare the activity of different enzymatic systems. Note that the activity of enzymes from different sources or batches may alter, therefore tuning the final concentrations may be necessary.

HRP assay. The guaiacol assay was employed to determine the HRP activity of the biocatalytic materials and to calculate the characteristic kinetic data[6]. Samples were prepared by mixing 240 μL of enzyme or enzyme containing composite of 5 mg/L loading, 1.872 mL of phosphate buffer (12.9 mM and pH 7) and 240 μL of aqueous guaiacol solution at different concentrations (the final concentrations of guaiacol were varied between 2 mM and 40 mM). The dispersions or solutions were thoroughly mixed by vortex. Then, 48 μL of 135 mM hydrogen peroxide solution was added and the evolution of the absorbance at 470 nm was followed[7]. The absorbance values were plotted as a function of the reaction time and the linear part of the graph (excluding the saturation and possible initial mixing stages) was used to extract the reaction rate (v) at a certain substrate concentration (S). Eventually, the obtained v values were plotted as a function of S and fitted with the Michaelis-Menten model using the following relation[8]:

$$\boldsymbol{v = \frac{v_{max} S}{K_M + S}} \qquad (3)$$

where v_{max} is the maximum rate of the enzymatic reaction, when the enzyme is fully saturated by the substrate and K_M is the Michaelis constant. Since the aim of the research was possible biomedical applications of the antioxidant compounds, it is important to know the enzyme activity at low substrate concentrations that are usual for physiological conditions. This is well described by the catalytic efficiency (k_{cat}/K_M), where k_{cat} corresponds to the turnover number (number of catalytic conversions that the enzyme is able to perform at a certain substrate concentration per second). If one assumes that the reaction follows the Michaelis-Menten kinetics with a steady-state condition (the concentration of the enzyme-substrate complex remains the same during the reaction) and that a total enzyme concentration (E_{tot}) is equal to the free enzyme concentration (E) due to the low amount of substrate ($S \ll K_M$), using $E_{tot} = E + S \approx E$ relation, one can derive the following equation that determines the rate of the reaction at physiological conditions (v_0):

$$v_0 = \frac{k_{cat}}{K_M} E_{tot} S \tag{4}$$

This equation shows the importance of the catalytic efficiency (k_{cat}/K_M) and the main reason why it was calculated and compared among different enzymatic systems. In order to obtain it, K_M was determined from the Michaelis-Menten fit and k_{cat} directly from the maximum reaction rate (v_{max}) as:

$$v_{max} = k_{cat} E_{tot} \tag{5}$$

Guaiacol assay used for cellular uptake measurements. The supernatant liquids (about 1.2 mL in each case) after cell lysis were transferred into a cuvette. Thereafter, 912 μL of phosphate buffer (131.6 mM and pH 7), 240 μL of 0.1 M guaiacol solution and 48 μL of 135 mM hydrogen peroxide solution was added to the samples. After a quick mixing on a vortex, the absorbance change at 470 nm was measured for 400 s. The color changes are demonstrated in Fig. S4. The samples were probed at one final guaiacol concentration (10 mM, to detect coloration), thus kinetic data were not gained in this case. The above conditions led to an average error of 4%.

Detection of intracellular oxidative stress. After a total 60 min incubation time with the active material (from which 20 min was loading with the oxidative stress sensor H_2DCFDA as well), the cover glass carrying the cells was transferred to the microscope in a perfusion cell. The initial perfusion was with HEPES buffer only (pH 7.5, 2 min), which was followed by the perfusion of 50 μM menadione in the same HEPES buffer. The fluorescent response was followed for 200 s. Each detection was 5 s apart, compiling up to 48 cycles. The excitation and emission wavelengths were 495 and 525 nm, respectively. After the reaction terminated, the response activities were normalized and drift corrected.

Cell viability in the presence of the composite materials. After incubation with the **CASCADE** material for 60 min, the liquid was removed by a pipette and the cover glass with the cells was washed with the assay buffer included in the apoptosis/necrosis kit. The loading dye solution was prepared by adding 1 μL CytoCalcein Violet 450, 2 μL Apopxin Green and 1

μL 7-AAD dye to 200 μL assay buffer. In this order, the dyes indicate live, apoptotic and necrotic fluorescing cells. The cells were loaded with the dye mix solution for 20 min at 37 °C, 5 v% CO_2 in air. After loading, the dyes were removed with a pipette, the cells were washed again and transferred for microscopic imaging. Cell emission color indicates the compatibility of the cells with the composite material. Excitation and emission (in brackets) wavelengths were the following (in nm): CytoCalcein Violet 450 (405/450) Apopxin Green (490/525) and 7-AAD (550/650). The biochemical background of the dye fluorescence is as follows: CytoCalcein Violet is sequestered in the cytoplasm of live cells to yield the fluorescent calcein dye, Apopxin Green binds with phosphatidylserine, an indicator molecule of apoptosis to form the fluorescent dye, 7-AAD, a healthy cell membrane impermeable dye labels the nucleus of a severely damaged necrotic (or late apoptotic) cell. Note that tiny green spots outside the cells correspond to measurement artifacts.

References

1. Xu, Z. P., *et al.* Stable suspension of layered double hydroxide nanoparticles in aqueous solution. *J. Am. Chem. Soc.* **128**, 36-37 (2006).
2. Pavlovic, M., Rouster, P. & Szilagyi, I. Synthesis and formulation of functional bionanomaterials with superoxide dismutase activity. *Nanoscale* **9**, 369-379 (2017).
3. Holthoff, H., Egelhaaf, S. U., Borkovec, M., Schurtenberger, P. & Sticher, H. Coagulation rate measurements of colloidal particles by simultaneous static and dynamic light scattering. *Langmuir* **12**, 5541-5549 (1996).
4. Bradford, M. M. Rapid and sensitive method for quantitation of microgram quantities of protein utilizing principle of protein-dye binding. *Anal. Biochem.* **72**, 248-254 (1976).
5. Beaucham, C. & Fridovich, I. Superoxide dismutase - improved assays and an assay applicable to acrylamide gels. *Anal. Biochem.* **44**, 276-287 (1971).
6. Maehly, A. C. & Chance, B. The assay of catalases and peroxidases. *Methods Biochem. Anal.* **1**, 357-424 (1954).
7. Doerge, D. R., Divi, R. L. & Churchwell, M. I. Identification of the colored guaiacol oxidation product produced by peroxidases. *Anal. Biochem.* **250**, 10-17 (1997).
8. Johnson, K. A. & Goody, R. S. The original Michaelis constant: Translation of the 1913 Michaelis-Menten paper. *Biochemistry* **50**, 8264-8269 (2011).
9. Evans, D. G. & Slade, R. C. T. Structural aspects of layered double hydroxides. In: *Layered Double Hydroxides* (eds Duan X, Evans DG) (2006).
10. Kuang, Y., *et al.* Morphologies, preparations and applications of layered double hydroxide micro-/nanostructures. *Materials* **3**, 5220-5235 (2010).

Supplementary Table S1 Extracted parameters of the Mann-Whitney U-test for ROS inhibition measurements.[a] The fluorescent response of H_2DCFDA dye was evaluated and the samples were tested against the control experiments. The applied significance level (p value) was 5%.

	**Control**	**5 mg/L**	**10 mg/L**	**20 mg/L**
Median of normalized fluorescent response	4.91±2.62	4.45±0.51	2.26±0.22	1.34±0.40
U value	–	43	12	4
Sample size	8	13	29	13
p value / %	–	53.4	<0.01	<0.01
Significantly different from control?	–	No	Yes	Yes

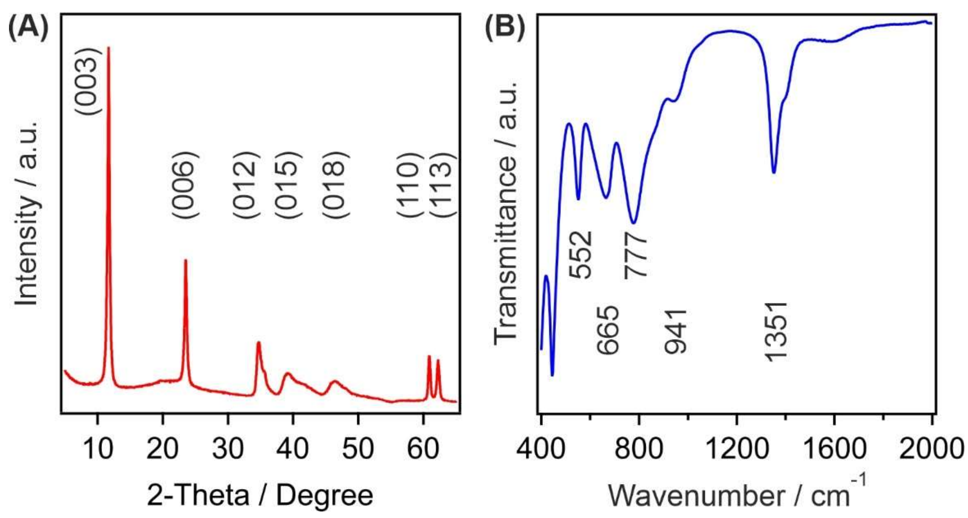

Supplementary Fig. S1 Structural features of the LDH carrier. (A) X-Ray diffraction pattern of the LDH with the Miller indices indicated. (B) Infrared spectrum with the wavenumbers of the characteristic vibrations shown. Comparing to the literature data,[9,10] these results support the formation of single-phase LDH materials of intercalated carbonate anions.

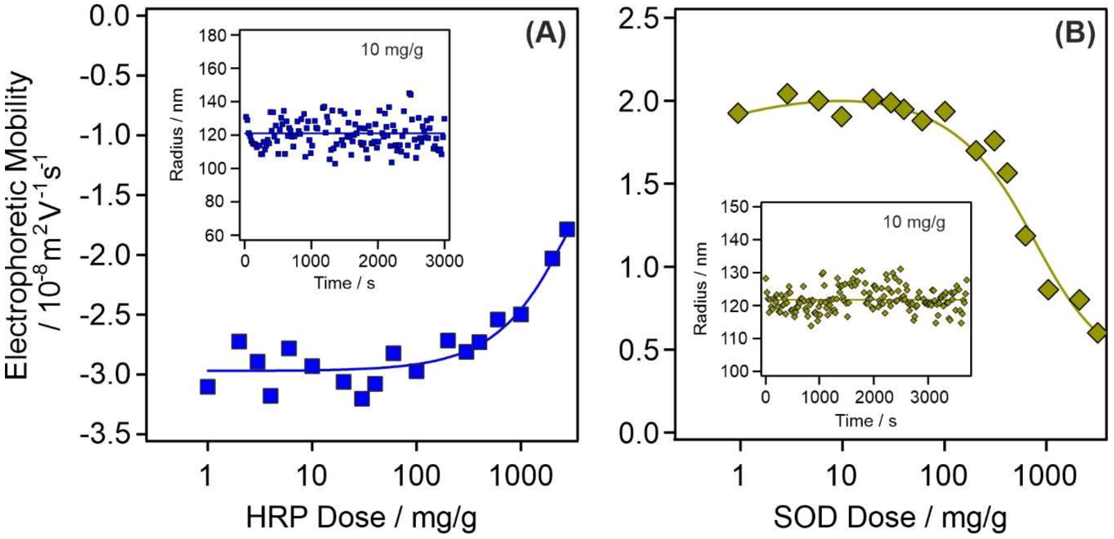

Supplementary Fig. S2 Influence of enzyme adsorption on the surface charge and aggregation of the carrier particles. (A) Electrophoretic mobilities of the LDH-HEP composite at different HRP doses. The inset shows hydrodynamic radii measured in time-resolved DLS experiments at 10 mg/g enzyme dose. (B) Electrophoretic mobilities of the LDH-HEP-HRP-PLL particles at different SOD doses. The inset shows hydrodynamic radii measured in time-resolved DLS experiments at 10 mg/g enzyme dose. These results confirm that enzyme adsorption at 10 mg/g dose does not change the surface charge density significantly and that the LDH-HEP-HRP and LDH-HEP-HRP-PLL-SOD particles form stable dispersions. The electrophoretic mobilities were determined with $\pm 10^{-9}$ $m^2V^{-1}s^{-1}$ precision and the maximum standard deviation in the hydrodynamic radii was ±5 nm.

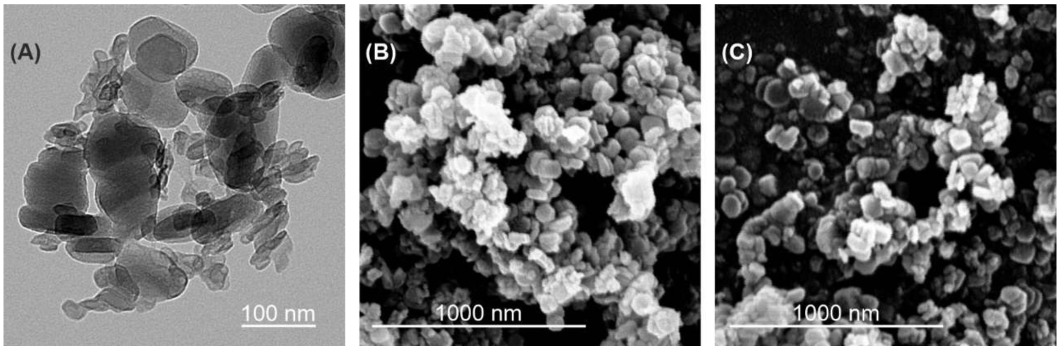

Supplementary Fig. S3 Electron microscopy images of LDH. (A) TEM and (B)-(C) SEM pictures of the bare LDH carrier particles.

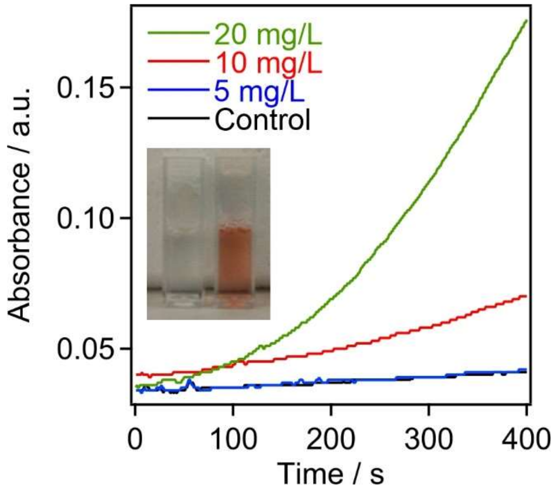

Supplementary Fig. S4 Photometric guaiacol assay of cell lysate to assess take-up of CASCADE by HeLa cells. Photometric response of guaiacol assay at 470 nm. The inset photograph shows the visual proof for HRP inactivity (left, control) and HRP activity (right, 20 mg/L **CASCADE**). The orangish substance is the oxidized form of guaiacol[7], as a result of the presence of a HRP-active material in the cuvette.

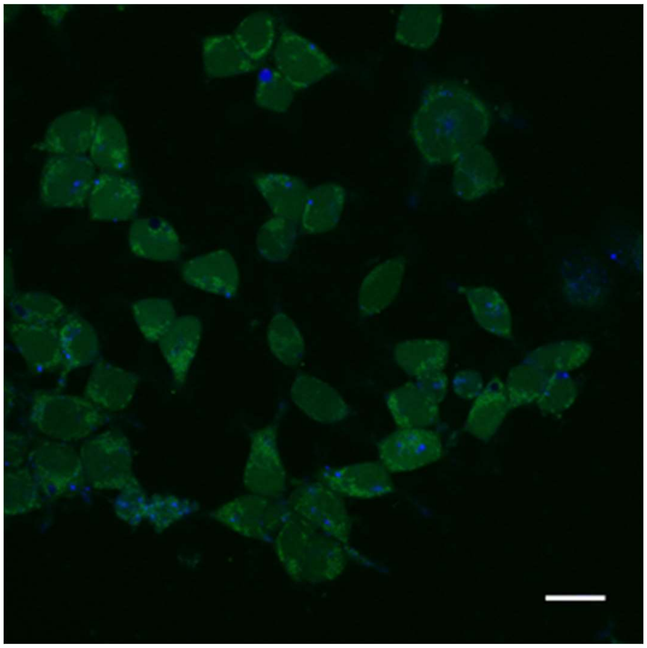

Supplementary Fig. S5 Viability study on HeLa cells. Extensive apoptosis (programmed cell death) is indicated by the green color at 40 mg/L CASCADE concentration. The scale bar represents 20 µm dimension.
